# A large-scale meta-analysis to refine colorectal cancer risk estimates associated with *MUTYH* variants

**DOI:** 10.1038/sj.bjc.6605966

**Published:** 2010-11-09

**Authors:** E Theodoratou, H Campbell, A Tenesa, R Houlston, E Webb, S Lubbe, P Broderick, S Gallinger, E M Croitoru, M A Jenkins, A K Win, S P Cleary, T Koessler, P D Pharoah, S Küry, S Bézieau, B Buecher, N A Ellis, P Peterlongo, K Offit, L A Aaltonen, S Enholm, A Lindblom, X-L Zhou, I P Tomlinson, V Moreno, I Blanco, G Capellà, R Barnetson, M E Porteous, M G Dunlop, S M Farrington

**Affiliations:** 1 Colon Cancer Genetics Group and Academic Coloproctology, MRC Human Genetics Unit, Institute of Genetics and Molecular Medicine, University of Edinburgh, Edinburgh, UK; 2 Public Health Sciences, University of Edinburgh, Teviot Place, Edinburgh, UK; 3 Southeast of Scotland Clinical Genetic Services, University of Edinburgh, Edinburgh, UK; 4 Section of Cancer Genetics, Institute of Cancer Research, Sutton, Surrey, UK; 5 Ontario Familial Colorectal Cancer Registry, Toronto, Ontario, Canada; 6 Samuel Lunenfeld Research Institute, Mount Sinai Hospital, Toronto, Canada; 7 Centre for Molecular, Environmental, Genetic and Analytic Epidemiology, The University of Melbourne, Victoria, Australia; 8 Strangeways Research Laboratory, Department of Oncology and Department of Public Health and Primary Care, University of Cambridge, Cambridge, UK; 9 CHU de Nantes, pôle de Biologie, service de Génétique Médicale, 9 quai Moncousu, Nantes, 44093 cedex 1, France; 10 unité de Génétique Constitutionnelle, Institut Curie, service de Génétique Oncologique, 26 rue d’Ulm, Paris, 75248 cedex 05, France; 11 University of Chicago, 900 East 57th Street, Chicago, IL 60637, USA; 12 IFOM, Fondazione Istituto FIRC di Oncologia Molecolare, and Unit of Genetic Susceptibility to Cancer, Department of Experimental Oncology and Molecular Medicine, Fondazione IRCCS Istituto Nazionale dei Tumori, Milan, Italy; 13 Clinical Cancer Genetics, Memorial Sloan-Kettering Cancer Centre, New York, USA; 14 Department of Medical Genetics, Biomedicum Helsinki, University of Helsinki, PO Box 63 (Haartmaninkatu 8), FIN-00014, Finland; 15 Department of Molecular Medicine and Surgery Karolinska Institute and Department of Clinical Genetics Karolinska University Hospital, Stockholm, Sweden; 16 Molecular and Population Genetics Laboratory, London Research Institute, Cancer Research UK, London, UK; 17 Catalan Institute of Oncology-ICO, IDIBELL and University of Barcelona, Av Gran Via 199, L'Hospitalet, Barcelona 08907, Spain

**Keywords:** colorectal cancer, base excision repair, *MUTYH*, carrier risk estimates, meta-analysis

## Abstract

**Background:**

Defective DNA repair has a causal role in hereditary colorectal cancer (CRC). Defects in the base excision repair gene *MUTYH* are responsible for *MUTYH*-associated polyposis and CRC predisposition as an autosomal recessive trait. Numerous reports have suggested *MUTYH* mono-allelic variants to be low penetrance risk alleles. We report a large collaborative meta-analysis to assess and refine CRC risk estimates associated with bi-allelic and mono-allelic *MUTYH* variants and investigate age and sex influence on risk.

**Methods:**

*MUTYH* genotype data were included from 20 565 cases and 15 524 controls. Three logistic regression models were tested: a crude model; adjusted for age and sex; adjusted for age, sex and study.

**Results:**

All three models produced very similar results. *MUTYH* bi-allelic carriers demonstrated a 28-fold increase in risk (95% confidence interval (CI): 6.95–115). Significant bi-allelic effects were also observed for G396D and Y179C/G396D compound heterozygotes and a marginal mono-allelic effect for variant Y179C (odds ratio (OR)=1.34; 95% CI: 1.00–1.80). A pooled meta-analysis of all published and unpublished datasets submitted showed bi-allelic effects for *MUTYH*, G396D and Y179C (OR=10.8, 95% CI: 5.02–23.2; OR=6.47, 95% CI: 2.33–18.0; OR=3.35, 95% CI: 1.14–9.89) and marginal mono-allelic effect for variants *MUTYH* (OR=1.16, 95% CI: 1.00–1.34) and Y179C alone (OR=1.34, 95% CI: 1.01–1.77).

**Conclusions:**

Overall, this large study refines estimates of disease risk associated with mono-allelic and bi-allelic *MUTYH* carriers.

Oxidative damage to DNA occurs with cell proliferation and increases with age. Certain organs such as the gut are heavily exposed to oxidising agents, which impacts on carcinogenic potential. Dysfunction of base excision repair, the major pathway for repairing oxidative damage, has been implicated as a risk factor for the development of multiple colorectal adenomas and colorectal cancer (CRC; Al-Tassan *et al*, 2002; Croitoru *et al*, 2004; Farrington *et al*, 2005). Bi-allelic mutations of the *MUTYH* gene seem to be responsible for a high proportion of the multiple adenoma phenotype families (termed *MUTYH*-associated polyposis (MAP)) unaccounted for by germline *APC* mutations (Al-Tassan *et al*, 2002; Sampson *et al*, 2003; Sieber *et al*, 2003; Gismondi *et al*, 2004; Venesio *et al*, 2004; Nielsen *et al*, 2009) and predispose to CRC *per se* (Enholm *et al*, 2003; Croitoru *et al*, 2004; Fleischmann *et al*, 2004; Kambara *et al*, 2004; Wang *et al*, 2004; Farrington *et al*, 2005; Peterlongo *et al*, 2005; Zhou *et al*, 2005; Moreno *et al*, 2006; Tenesa *et al*, 2006; Webb *et al*, 2006; Küry *et al*, 2007; Cleary *et al*, 2009; Lubbe *et al*, 2009). Although an increased CRC risk associated with bi-allelic *MUTYH* mutations is incontrovertible, the risk associated with one *MUTYH* mutant allele is controversial (Croitoru *et al*, 2004; Farrington *et al*, 2005; Jenkins *et al*, 2006; Tenesa *et al*, 2006; Webb *et al*, 2006; Cleary *et al*, 2009; Jones *et al*, 2009; Lubbe *et al*, 2009). A statistically significant or close to significant effect, for a *MUTYH* mono-allelic effect, has been reported in different studies with possible age specific effects present, but the rarity of the alleles associated with the small increased risk for CRC have made it difficult to replicate study findings. A recent risk analysis of MAP family members agreed with previous family based findings (Jenkins *et al*, 2006) that mono-allelic carriers are at a two-fold increase in risk of CRC (Jones *et al*, 2009) providing further evidence of a mono-allelic effect of the gene. However, family based studies can be subject to ascertainment bias and any mono-allelic effect could potentially be modified by other inherited factors, including alleles at other loci. Furthermore, environmental risk factors also show familial aggregation and hence, studies in which there has been selection of cases based on family history may be confounded. Bi-allelic carriers may develop CRC because of the predominant effect of *MUTYH*, whereas the environmental effect is greater in affected siblings with mono-allelic mutations but the risk is ascribed to the *MUTYH* allele. Thus further work is required to resolve the mono-allelic carrier risk question.

To clarify the role of *MUTYH* in disease risk, we initiated a multi-centre collaboration allowing large-scale meta-analysis of the individual *MUTYH* variants, with special interest in determining if there were age and sex-specific effects on CRC association with *MUTYH* variants (Farrington *et al*, 2006). In this study, we present the results of this collaborative meta-analysis.

## Subjects and methods

### Participating studies

Relevant case–control studies to be invited for inclusion in the meta-analysis of the effect of *MUTYH* on CRC risk were identified by a literature search in the ISI Web of Science (http://wok.mimas.ac.uk) and PUBMED bibliographic databases (http://www.ncbi.nlm.nih.gov/pubmed/), using the search terms ‘MYH or *MUTYH* and CRC’. In the initial search 55 studies were identified and eight of these were considered for our study (Enholm *et al*, 2003; Croitoru *et al*, 2004; Fleischmann *et al*, 2004; Kambara *et al*, 2004; Wang *et al*, 2004; Farrington *et al*, 2005; Peterlongo *et al*, 2005; Zhou *et al*, 2005). The inclusion criteria were as follows: the patients had to be diagnosed with CRC and the studies had to have genotype data for both cases and controls. Ten additional studies were identified during the progress of the project – Webb *et al* (2006), Moreno *et al* (2006), Küry *et al* (2007), Cleary *et al* (2009), Lubbe *et al* (2009); and unpublished data from Koessler T and Pharoah PD; and Tomlinson – personal communication. Colebatch *et al* (2006); Balaguer *et al* (2007); Avezzù *et al* (2008) were used in the pooled meta-analysis of all available published and unpublished datasets.

The principal investigators (PIs) of the selected studies were contacted and were asked to participate by providing a minimum dataset including variables necessary for the analysis (Supplementary Box 1: Study questionnaire; Supplementary Table 1: Data extraction table). In cases, in whom PIs failed to respond to our invitation to participate, reminder letters were despatched. It was not possible to include data from the following studies in the logistic regression analyses because (i) data was only available for cases that were heterozygous or homozygous for a *MUTYH* mutation (Enholm *et al*, 2003); (ii) co-variate data were only available for cases, as controls were anonymous blood donors (Zhou *et al*, 2005; Tomlinson, unpublished data); (iii) failure to communicate with us (Kambara *et al*, 2004 and Wang *et al*, 2004). The study by Fleischmann *et al* (2004) and Webb *et al* (2006) were not used because they had been superseded by a later study (Lubbe *et al*, 2009), which was included.

### Statistical analysis

Data from all collaborating centres were checked for completeness, coded and merged to form a core database. *MUTYH* defects were considered pathogenic only if there was published evidence of their pathogenicity. Individuals reported to have two defects of *MUTYH* in the original report were classified as mutated/mutated (MM), those with one defect as wild type/mutant (WM) and those with no mutation as wild type/wild type (WW). Descriptive statistics were produced on all subject characteristics, risk factors and event data. All populations described in the case–control studies were tested for Hardy–Weinberg equilibrium in controls and the genotype distributions between all groups were compared by *χ*^2^-test.

Three logistic regression models were applied to address confounding co-variates (model I: crude, model II: including co-variates for age and sex, model III: including co-variates for age, sex and study) on the combined datasets investigating the effect of *MUTYH* defects (WM *vs* WW and MM *vs* WW) as well as of the individual mutations Y179C (c.536A>G/p.Tyr179Cys; AA=WW, GG=MM) and G396D (c.1187G>A/p.Gly396Asp; GG=WW, AA=MM; previously known as Y165C and G382D), to identify any variant specific associations. The three logistic regression models were applied after sex and age (over 55 years and under or equal 55 years) stratification as previously described (Farrington *et al*, 2005), to assess the effect of age and sex on the association of the variants with disease risk. In all the studies, interaction associations between the *MUTYH* variants and study code (for each individual study) were estimated and similarly between *MUTYH* variants and hormone replacement therapy (HRT) among female participants in three studies (the Scottish SOCCS studies and the studies – Croitoru *et al*, 2004; Cleary *et al*, 2009). Association between both genetic (i.e., one of the *MUTYH* mutations) and the study code or environmental factor (i.e., HRT) and disease was assessed and interaction was tested by fitting interactive and nested multiplicative models. To assess for any small study effects, we performed Funnel plot analysis and tested for significance using the Harbord test.

Finally, the relationship between the genotype and CRC was analysed by meta-analysis, combining the effect estimates of all published and unpublished datasets.

All statistic analyses were conducted using Intercooled STATA version 10.0 (Stata Corp, College Station, TX, USA). For the logistic regression analyses, it is necessary to add a whole number to any fields containing 0 (see model I^a^ in Table 2), which reduces the final OR value, however, by using the META command in the STATA meta-analysis programme, a lower value can be added (0.5 as indicated by model I^b^ in Table 2) thereby giving a more accurate assessment of risk. However, this is a grouped analysis and therefore cannot be adjusted for confounding co-variates, such as age/sex and study as in models II and III. To account for multiple testing we applied the Bonferroni correction method, and the *P*-value threshold for significance was estimated to be 0.003.

## Results

Table 1 details summary data from the studies included in our combined analysis (comprising a total of 20 565 cases and 15 524 controls). The two variant alleles are rare with G396D variant allele having a frequency of 0.007 in controls and the Y179C variant allele a frequency of 0.002. Tests for deviation from Hardy–Weinberg equilibrium in controls were *P*=0.99 and *P*<0.00005 for G396D and Y179C variants, respectively.

### Bi-allelic effect of *MUTYH*

All three models of the logistic regression analysis gave consistent results and so the results of the crude analysis (model I) are described below and presented in Table 2; results of the other two models can be found in Supplementary Table 2. Bi-allelic carriers for the MM genotype of the combined *MUTYH* defects, G396D and Y179C/G396D compound heterozygotes were associated with a significant increase in CRC risk (odds ratio (OR)=28.3, 95% confidence limits (CIs): 6.95–115; 23.1 (95% CI: 3.15–169) and 21.6 (95% CI: 2.94–159), respectively). These risks are conservative, concentrating on the significant logistic regression results – model I^b^ results presented in Table 2 are likely a better reflection of risk and tend to be two-fold higher. There was a greater CRC risk for the MM genotype for the earlier age individuals when compared with the older age group (OR=36.2 (95% CI: 4.98–263) for ⩽55 years compared with 11.6 (95% CI: 2.77–48.2) for >55 years). However, their CIs overlapped and the results were not statistically significantly different. ANOVA analysis of mean age of carriers demonstrated that there are significant age differences between cases and controls when considering MM genotype carriers and Y179C bi-allelic carriers but not for G396D carriers (*P*<0.0005, *P*<0.0005 and *P*=0.27, respectively – Supplementary Table 3). Indeed there is a significant age difference between mean age of bi-allelic Y179C and G396D carriers (48.9 *vs* 56.7, respectively, *P*=0.003 based on *t*-test – Supplementary Table 4).

### Colorectal cancer risk associated with mono-allelic *MUTYH* mutations

The results of the combined analysis demonstrate that there are no significant mono-allelic effects for either G396D or for combined *MUTYH* variants (Table 2). However, the specific Y179C variant was shown to increase risk of disease in the heterozygous state (OR=1.34; (95% CI: 1.00–1.80)) in the whole sample set and also when stratified by sex, male sex demonstrated a mono-allelic effect (OR=1.70; (95% CI: 1.06–2.73)). However, after Bonferroni correction, these mono-allelic effects did not remain significant.

### The role of study population and HRT in modulating CRC risk

We hypothesised that origin of the data, that is, study population might modify the association between the genotype and CRC risk. However, there was no evidence for an interaction between study code and *MUTYH* genotypes (Supplementary Table 5). Similarly, HRT intake, a known risk factor for CRC (Chan *et al*, 2006; Theodoratou *et al*, 2008), might be influenced by genotype and therefore modulate female risk. Both the Scottish and Canadian datasets had recorded data on HRT intake and these were used to test for an interaction between HRT and *MUTYH* genotype. Across both datasets there was no evidence of any interaction between HRT and *MUTYH* genotype (Supplementary Table 6).

### Meta-analysis of published and unpublished datasets

**Figure 1:**
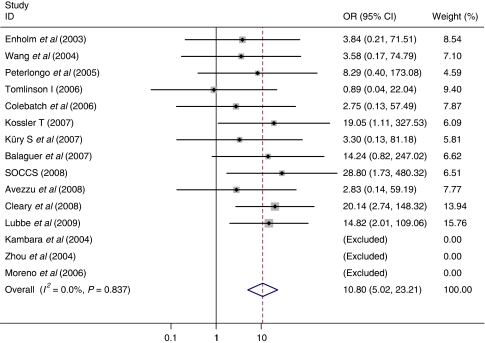
Meta-analysis of studies comparing *MUTYH* MM *vs* WW. SOCCS data include Farrington *et al* (2005), Tenesa *et al* (2006) and unpublished data from the SOCCS study obtained in 2008; Cleary data include Croitoru *et al* (2004); Lubbe data include Webb *et al* (2006) and Fleischmann *et al* (2004). Unpublished studies included are Tomilson I (2006) and Koessler T (2007).

**Figure 2:**
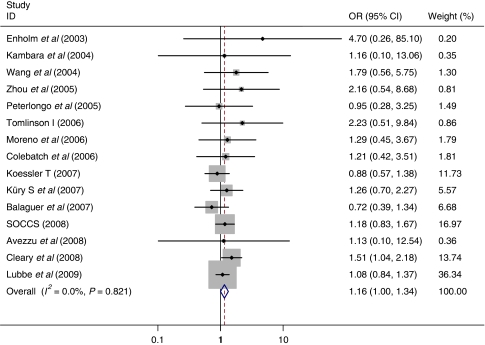
Meta-analysis of studies comparing *MUTYH* WM *vs* WW. SOCCS data include Farrington *et al* (2005), Tenesa *et al* (2006) and unpublished data from the SOCCS study obtained in 2008; Cleary data include Croitoru *et al* (2004); Lubbe data include Webb *et al* (2006) and Fleischmann *et al* (2004). Unpublished studies included are Tomilson I (2006) and Koessler T (2007).

The results of a meta-analysis of published and unpublished datasets submitted to us, estimating the effect of the *MUTYH* whole gene defects demonstrated a pooled fixed bi-allelic effect of 10.8 (95% CI: 5.02–23.2) for the MM and a pooled fixed mono-allelic effect of 1.16 (95% CI: 1.00–1.34) for WM genotype (Table 3; Figures 1 and 2). Analysis of the specific variants by pooled meta-analysis demonstrated bi-allelic effects for both G396D and Y179C (OR=6.47 (95% CI: 2.33–18.0) and OR=3.35 (95% CI: 1.14–9.89), respectively) and in agreement with the logistic regression analysis results, Y179C variant also demonstrated a very similar pooled fixed mono-allelic effect of 1.34 (95% CI: 1.01–1.77; Tables 4 and 5; Supplementary Figures 1–4).

### Assessment of study publication bias

Funnel plots for both the mono- and bi-allelic effect were created to assess whether study size was significantly influencing the results. These plots appeared asymmetric, but the Harbord's test for small study effect demonstrated that this was not statistically significant (Supplementary Figures 5 and 6).

## Discussion

This large meta-analysis study refines the estimates of CRC risk associated with mutations in the *MUTYH* gene to date. Bi-allelic carriers of the combined *MUTYH* mutations (MM) are associated with a 28-fold (95% CI: 6.95–115) increase in CRC risk from the logistic regression analysis. Bi-allelic carriers of the G396D variant and Y179C/G396D compound heterozygotes were also significantly associated with a similar increase in CRC risk (OR=23.1 (95% CI: 3.15–169) and 21.6 (95% CI: 2.94–159), respectively). Although the risk estimate was slightly lower from the overall larger pooled meta-analysis of published and unpublished datasets (OR=10.8 (95% CI: 5.02–23.2)), both G396D and Y179C variants demonstrated bi-allelic effects in this pooled analysis (OR=6.47 (95% CI: 2.33–18.0) and OR=3.35 (95% CI: 1.14–9.89), respectively). A marginal significant mono-allelic effect was demonstrated for the specific variant Y179C (OR=1.34 (95% CI: 1.00–1.80)) and indeed a marginally significant result was also observed in the pooled meta-analysis for *MUTYH* WM (OR=1.16 (95% CI: 1.00–1.34)) and Y179C variant alone, 1.34 (95% CI: 1.01–1.77). The increased bi-allelic risk of CRC varied when stratified for age and sex but none of the differences were significant, although when stratified by sex, males showed a marginal significant mono-allelic effect for Y179C (OR=1.70; 95% CI: 1.06–2.73). The results from this large dataset indicate that the two variants may be acting mechanistically differently; G396D appears to be a true example of recessive Mendelian disease, whereas the results for Y179C are more complex and there is therefore some argument against combining the two variants. However, the results from the Y179C/G396D compound heterozygotes analysis demonstrates an increase in risk similar to G396D bi-allelic carriers, suggesting that the two variants are complementary and analysis of combined *MUTYH* mutations as historically performed, appears appropriate to assess risk for the whole gene. The rarity of the Y179C allele has made it difficult to truly assess its effect on disease risk, however the large numbers analysed in this report have resulted in the demonstration that both bi-allelic and mono-allelic Y179C variants are associated with disease risk.

The study population did not appear to modulate disease risk and although the study replicated the reported decrease in disease risk in *MUTYH* wild-type females associated with HRT intake (Chan *et al*, 2006; Theodoratou *et al*, 2008), we found no interaction with the *MUTYH* gene and its variants. Therefore, it is unlikely that HRT intake is an explanation for any sex variation in risk and other genetic factors may be involved in modifying CRC risk.

Evidence of a mono-allelic *MUTYH* effect on CRC has been reported in several case–control studies (Croitoru *et al*, 2004; Wang *et al*, 2004; Farrington *et al*, 2005; Zhou *et al*, 2005; Tenesa *et al*, 2006; Cleary *et al*, 2009) and family-based studies (Jenkins *et al*, 2006; Jones *et al*, 2009), but not in other studies (Kambara *et al*, 2004; Webb *et al*, 2006; Balaguer *et al*, 2007; Lubbe *et al*, 2009). Our large meta-analysis has demonstrated a marginal significant association for the specific variant Y179C, highlighting the possible increased phenotypic severity of this allele. This is in agreement with other studies and biochemical and model organism studies, which indicate that this variant shows an increased detrimental effect on protein function (Al-Tassan *et al*, 2002; Parker *et al*, 2005; Lubbe *et al*, 2009; Nielsen *et al*, 2009; D’Agostino *et al*, 2010). The pooled analysis of published studies and unpublished datasets submitted to us also indicated a marginally significant mono-allelic *MUTYH* effect, as well as a mono-allelic Y179C effect.

However, there are a number of caveats that need to be considered; if any of the studied datasets contain cases recruited because of the familial clustering of disease, there may be ascertainment bias, artificially inflating the number of *MUTYH* WM variant allele carriers; secondly the screening of the *MUTYH* gene has predominantly been performed on the two most common pathogenic variants Y179C and G396D – in some studies, the rest of the gene may be explored in cases with a heterozygous allele for these variants but not usually in the controls, hence there is an overall screening bias and bi-allelic carriers may well have been missed in both cases and controls.

The demonstration of a mono-allelic effect specifically for Y179C should be considered with further caution, as analysis of the control datasets for the Y179C allele demonstrated that it was not in Hardy–Weinberg equilibrium. This may be because of several factors, the rarity of the allele and the fact that both female control subjects with bi-allelic mutations carry Y179C variants. One of these control subjects was shown to have polyps on colonoscopy (Cleary *et al*, 2009) and may therefore be considered a case. The other is relatively young, less than 60 years old (Lubbe *et al*, 2009), so potentially may develop cancer over the next few years. However, in this large dataset, we have also shown that bi-allelic carriers of Y179C predisposes to an earlier onset of disease than G396D, consistent with previous reports (Lubbe *et al*, 2009; Nielsen *et al*, 2009) and highlights a severer disease phenotype of this variant.

In conclusion, inactivation of the *MUTYH* gene is a recessive risk factor for CRC, with possible modifying effects indicated by increased risk in cases with early age of onset, although not significantly different in the current dataset. An increased risk associated with mono-allelic *MUTYH* mutation is indicated, albeit small and not currently clinically relevant, and likely specific for the variant Y179C. Despite the size of this study it has not been possible to definitively establish whether there are significant age and sex effects of increasing disease risk for G396D and Y179C carriers. The evidence presented raises the possibility of a mono-allelic effect for Y179C, but the effect is low (OR 1.34; 95% CI: 1.00–1.80) and is sensitive to variations in population allele frequency because of the rarity of the variant (allele frequency 0.002), as well as potential issues of subgroup analysis and multiple testing (indeed the overall significance is lost after Bonferoni correction). Nonetheless, it does appear that this study is the first to demonstrate that the Y179C variant does impart an increased risk of CRC.

## Figures and Tables

**Table 1:** Summary table^a^

		**G396D**			**Y179C**			**Genotype**			**Age**	**Sex**		**Ethnicity**		
**Study**	**Number**	**GG**	**GA**	**AA**	**AA**	**AG**	**GG**	**WW**	**WM**	**MM**	***n* (%)**	**Mean (SD)**	***n* (%)**	**Males (%)**	***n* (%)**	**Caucasian/White (%)**
All studies																
*Cases*	20 565	19 966	324	33	20 260	154	11	19 783	417	76	20 555 (99.9)	59.5 (10.1)	20 555 (99.9)	11 668 (56.8)	5374 (26.1)	5030 (93.6)
*Controls*	15 524	15 145	211	0	15 074	68	2	14 723	280	2	15 287 (98.5)	59.0 (10.9)	15 521 (99.9)	6845 (44.1)	5597 (36.1)	4796 (85.7)

Croitoru *et al* (2004)																
*Cases*	1238	1209	25	4	1223	13	2	1197	29	12	1236 (99.8)	60.1 (8.7)	1236 (99.8)	544 (44.0)	Missing	Missing
*Controls*	1255	1238	17	0	1251	4	0	1234	21	0	1244 (99.1)	63.6 (8.6)	1252 (99.8)	702 (56.1)	Missing	Missing

Peterlongo *et al* (2005)																
*Cases*	585	567	3	1	569	3	0	552	4	2	584 (99.8)	62.1 (13.1)	584 (99.8)	319 (54.6)	585 (100)	251 (42.9)
*Controls*	1158	1040	5	0	1039	2	0	923	7	0	1148 (99.1)	53.9 (11.5)	1156 (99.8)	240 (20.8)	1158 (100)	363 (31.3)

SOCCS (includes data from Farrington *et al* (2005), Tenesa *et al* (2006) and unpublished SOCCS prospective samples)																
*Cases*	3278	3086	55	8	3192	23	0	3038	71	12	3277 (100)	59.5 (11.3)	3278 (100)	1883 (57.4)	3278 (100)	3268 (99.7)
*Controls*	3318	3239	43	0	3061	15	0	2993	57	0	3313 (99.8)	61.4 (10.9)	3318 (100)	1861 (56.1)	3318 (100)	3312 (99.8)

Moreno *et al* (2006)																
*Cases*	356	343	8	1	348	0	0	336	8	1	356 (100)	66.5 (11.7)	356 (100)	217 (61.0)	Missing	Missing
*Controls*	297	285	7	0	291	0	0	283	7	0	297 (100)	65.3 (12.5)	297 (100)	158 (53.2)	Missing	Missing

Koessler T, (unpublished data obtained in 2007)																
*Cases*	2262	2215	31	2	2227	19	1	2198	37	9	2253 (99.6)	59.1 (8.1)	2262 (100)	1287 (57.1)	Missing	Missing
*Controls*	2253	2217	31	0	2236	11	0	2204	42	0	2053 (91.1)	53.4 (7.6)	2253 (100)	949 (42.0)	Missing	Missing

Küry *et al* (2007)																
*Cases*	1025	1003	22	0	1021	4	0	999	25	1	1023 (99.8)	68.7 (9.9)	1025 (100)	632 (61.7)	1025 (100)	1025 (100)
*Controls*	1121	1105	16	0	1117	4	0	1100	21	0	1121 (100)	61.9 (10.0)	1121 (100)	609 (54.3)	1121 (100)	1121 (100)

SOCCS retrospective cases (unpublished data obtained in 2008)																
*Cases*	486	403	6	2	446	2	3	391	6	4	486 (100)	54.7 (17.2)	479 (98.6)	240 (50.1)	486 (100)	486 (100)
*Controls*	0	0	0	0	0	0	0	0	0	0	0		0		0	

Cleary *et al* (2009)																
*Cases*	2076	2023	33	5	2053	7	1	2029	38	9	2070 (99.7)	56.6 (10.8)	2076 (100)	1114 (53.7)	Missing	Missing
*Controls*	1049	1032	17	0	1042	6	1	1024	24	1	1047 (99.8)	56.4 (11.3)	1049 (100)	444 (42.3)	Missing	Missing

Lubbe *et al* (2009) b																
*Cases*	9268	9117	141	10	9181	83	4	9043	198	27	9268 (100)	59.0 (8.5)	9268 (100)	5432 (58.6)	Missing	Missing^c^
*Controls*	5064	4989	75	0	5037	26	1	4962	101	1	5064 (100)	59.2 (10.8)	5064 (100)	1882 (37.2)	Missing	Missing^c^

Abbreviations: MM=mutated/mutated; WM=wild type/mutant; WW=wild type/wild type.

a This table presents the raw data sent to us by each group that was then included in the analyses. G396D and Y179C are looked at independently and any compound bi-allelic carriers are presented as heterozygotes for each variant; MUTYH genotype data also includes other pathogenic mutations; unpublished data are included in Peterlonogo P and Moreno V, SOCCS prospective and Küry S.

b Includes data from Webb *et al* (2006) and Fleischmann *et al* (2004).

c All the cases and controls were UK residents and of European ancestry (self-reported).

**Table 2:** Logistic regression analysis of the combined datasets; G396D analysis was conducted for individuals that were Y179C AA; Y179C analysis was conducted for individuals that were G396D GG; combined genotype analysis was conducted for individuals with data for both Y179C and G396D

			**Model I** * ^,^ a			**Model I** b		
**Gene**	**Cases**	**Controls**	**OR**	**95% CI**	***P*-value**	**OR**	**95% CI**	***P*-value**
*G396D* c								
*Whole sample*								
GG	19 767	14 723	1.00					
GA	292	210	1.04	0.87, 1.24	0.70			
AA	31	0	23.09	3.15, 169.15	0.002	46.93	2.87, 766.89	0.007
⩽*55 Years old*								
GG	6269	5270	1.00					
GA	88	77	0.96	0.71, 1.31	0.80			
AA	13	0	10.93	1.43, 83.57	0.02	22.70	1.35, 381.91	0.03
>*55 Years old*								
GG	13 498	9453	1.00					
GA	204	133	1.07	0.86, 1.34	0.52			
AA	18	0	12.60	1.68, 94.37	0.01	25.91	1.56, 430.03	0.02
*Males*								
GG	11 229	6460	1.00					
GA	161	95	0.98	0.76, 1.26	0.85			
AA	15	0	8.62	1.14, 65.25	0.04	17.84	1.07, 298.11	0.04
*Females*								
GG	8528	8259	1.00					
GA	131	115	1.10	0.86, 1.42	0.45			
AA	16	0	15.49	2.05, 116.86	0.008	31.96	1.92, 532.72	0.02
								
*Y179C* d								
*Whole sample*								
AA	19 767	14 723	1.00					
AG	122	68	1.34	1.00, 1.80	0.05			
GG	11	2	4.10	0.91, 18.48	0.07	NA		
⩽*55 Years old*								
AA	6269	5270	1.00					
AG	35	25	1.18	0.70, 1.97	0.54			
GG	9	0	7.57	0.96, 59.74	0.06	15.97	0.93, 274.491	0.06
>*55 Years old*								
AA	13 498	9453	1.00					
AG	87	43	1.42	0.98, 2.04	0.06			
GG	2	2	0.70	0.10, 4.97	0.72	NA		
*Males*								
AA	11 229	6460	1.00					
AG	68	23	1.70	1.06, 2.73	0.03			
GG	8	0	4.60	0.58, 36.80	0.15	9.78	0.56, 169.48	0.12
*Females*								
AA	8528	8259	1.00					
AG	54	45	1.16	0.78, 1.73	0.46			
GG	3	2	1.45	0.24, 8.70	0.68	NA		
								
*Genotype* e								
*Whole sample*								
WW	19 767	14 723	1.00					
WM	418	280	1.11	0.95, 1.29	0.17			
MM	76	2	28.30	6.95, 115.26	3.1 × 10^−6^	NA		
G396D AA	31	0	23.09	3.15, 169.15	0.002	46.93	2.87, 766.89	0.007
Y179C GG	11	2	4.10	0.91, 18.48	0.07	NA		
Compound heterozygous^f^	29	0	21.60	2.94, 158.58	0.003	43.95	2.69, 719.26	0.008
								
⩽*55 Years old*								
WW	6269	5270	1.00					
WM	124	104	1.00	0.77, 1.30	0.99			
MM	43	0	36.15	4.98, 262.57	0.0004	73.14	4.50, 1188.3	0.003
G396D AA	13	0	10.93	1.43, 83.57	0.02	22.70	1.35, 381.91	0.03
Y179C GG	9	0	7.57	0.96, 59.74	0.06	15.97	0.93, 274.49	0.06
Compound heterozygous	17	0	14.29	1.90, 107.42	0.01	29.42	1.77, 489.38	0.02
								
>*55 Years old*								
WW	13 498	9453	1.00					
WM	294	176	1.17	0.97, 1.41	0.10			
MM	33	2	11.56	2.77, 48.17	0.001	NA		
G396D AA	18	0	12.60	1.68, 94.37	0.01	25.91	1.56, 430.03	0.02
Y179C GG	2	2	0.70	0.10, 4.97	0.72	NA		
Compound heterozygous	12	0	8.40	1.09, 64.64	0.04	17.51	1.04, 295.75	0.05
								
*Males*								
WW	11 229	6460	1.00					
WM	232	119	1.12	0.90, 1.40	0.31			
MM	36	0	20.71	2.84, 151.09	0.003	42.00	2.58, 684.38	0.009
G396D AA	15	0	8.62	1.14, 65.25	0.04	17.84	1.07, 298.11	0.04
Y179C GG	8	0	4.60	0.58, 36.80	0.15	9.78	0.56, 169.48	0.12
Compound heterozygous	12	0	6.90	0.90, 53.10	0.06	14.38	0.85, 242.96	0.06
								
*Females*								
WW	8528	8259	1.00					
WM	186	161	1.12	0.90, 1.38	0.30			
MM	40	2	19.37	4.68, 80.17	4.3 × 10^−5^	NA		
G396D AA	16	0	15.49	2.05, 116.86	0.008	31.96	1.92, 532.72	0.02
Y179C GG	3	2	1.45	0.24, 8.70	0.68	NA		
Compound heterozygous	17	0	16.46	2.19, 123.70	0.006	33.90	2.04, 563.74	0.01

Abbreviations: CI=confidence interval; MM=mutated/mutated; NA=not available; OR=odds ratio; WM=wild type/mutant; WW=wild type/wild type.

^*^Crude analysis.

a Estimated by adding one control with the variant genotype.

b Estimated using the meta command of STATA and for mathematical reasons, cells with zero frequencies were assumed to be 0.5 (as defaulted by the meta command).

c Analysis conducted only for the AA Y179C, that is, WW.

d Analysis conducted only for the GG G396D, that is, WW.

e Including subjects with data for both Y179C and G396D.

f This category includes 29 G396D GA and Y179C AG cases; 5 cases with either G396D GA or Y179C AG and any other pathogenic *MUTYH* mutation were excluded.

**Table 3:** Meta-analysis of studies^a^

	**Genotype (cases)**			**Genotype (controls)**			**WM *vs* WW**	**MM *vs* WW**
**Study**	**WW**	**WM**	**MM**	**WW**	**WM**	**MM**	**OR (95% CI)**	**OR (95% CI)**
Enholm *et al* (2003)	994	5	4	424	0	0	4.70 (0.26, 85.10)	3.84 (0.21, 71.51)
Kambara *et al* (2004)	90	2	0	52	—	0	1.16 (0.10, 13.06)	NA
Wang *et al* (2004)	432	10	2	309	4	0	1.79 (0.56, 5.75)	3.58 (0.17, 74.79)
Zhou *et al* (2005)	432	6	0	466	3	0	2.16 (0.54, 8.68)	NA
Peterlongo *et al* (2005)	549	4	2	911	7	0	0.95 (0.28, 3.25)	8.29 (0.40, 173.08)
Tomlinson I (2006)^b^	662	15	1	197	2	0	2.23 (0.51, 9.84)	0.89 (0.04, 22.04)
Moreno *et al* (2006)	323	9	0	278	6	0	1.29 (0.45, 3.67)	NA
Colebatch *et al* (2006)	859	11	2	473	5	0	1.21 (0.42, 3.51)	2.75 (0.13, 57.49)
Koessler T (2007)^c^	2198	37	9	2204	42	0	0.88 (0.57, 1.38)	19.05 (1.11, 327.53)
Küry *et al* (2007)	999	24	1	1100	21	0	1.26 (0.70, 2.27)	3.30 (0.13, 81.18)
Balaguer *et al* (2007)	1089	19	8	912	22	0	0.72 (0.39, 1.34)	14.24 (0.82, 247.02)
SOCCS (2008)^d^	3429	77	16	2993	57	0	1.18 (0.83, 1.67)	28.80 (1.73, 480.32)
Avezzù *et al* (2008)	435	2	2	246	1	0	1.13 (0.10, 12.54)	2.83 (0.14, 59.19)
Cleary *et al* (2009) e	3697	87	27	2758	43	1	1.51 (1.04, 2.18)	20.14 (2.74, 148.32)
Lubbe *et al* (2009) f	9043	198	27	4962	101	1	1.08 (0.84, 1.37)	14.82 (2.01, 109.06)
*M–H pooled effect (fixed)*	25231	506	101	18285	315	2	1.16 (1.00, 1.34)	10.80 (5.02, 23.21)
*P*-value							0.05	<0.0005
								
*Heterogeneity*								
*P*-value							0.82	0.84
*I*^2^ (95% CI)							0 (0, 54)	0 (0, 60)

Abbreviations: CI=confidence interval; MM=mutated/mutated; NA=not available; OR=odds ratio; WM=wild type/mutant; WW=wild type/wild type.

a This table presents the data as they were published. Two unpublished studies included.

b Unpublished data obtained in 2006.

c Unpublished data obtained in 2007.

d Includes data from Farrington *et al* (2005), Tenesa *et al* (2006) and unpublished data from the SOCCS study obtained in 2008.

e Includes data from Croitoru *et al* (2004).

f Includes data from Webb *et al* (2006) and Fleischmann *et al* (2004).

**Table 4:** Meta-analysis of studies for the G396D genotypes^a^

	**G396D (cases)**			**G396D (controls)**			**GA *vs* GG**	**AA *vs* GG**
**Study**	**GG**	**GA**	**AA**	**GG**	**GA**	**AA**	**OR (95% CI)**	**OR (95% CI)**
Enholm *et al* (2003)	994	4	1	424	0	0	3.84 (0.21, 71.51)	1.28 (0.05, 31.50)
Kambara *et al* (2004)	90	2	0	52	—	0	Not enough data available	NA
Wang *et al* (2004)	432	5	0	309	2	0	1.79 (0.35, 9.28)	NA
Zhou *et al* (2005)	432	1	0	466	1	0	1.08 (0.07, 17.30)	NA
Peterlongo *et al* (2005)	549	2	0	911	5	0	0.66 (0.13, 3.43)	NA
Tomlinson I (2006)^b^	662	9	1	197	2	0	1.34 (0.29, 6.25)	0.89 (0.04, 22.04)
Moreno *et al* (2006)	323	9	0	278	6	0	1.29 (0.45, 3.67)	NA
Colebatch *et al* (2006)	859	8	0	473	4	0	1.10 (0.33, 3.67)	NA
Koessler T (2007)^c^	2198	25	2	2204	31	0	0.81 (0.48, 1.37)	5.01 (0.24, 104.49)
Küry *et al* (2007)	999	21	0	1100	16	0	1.45 (0.75, 2.79)	NA
Balaguer *et al* (2007)	1089	15	1	912	20	0	0.63 (0.32, 1.23)	2.51 (0.10, 61.75)
SOCCS (2008)^d^	3429	56	8	2993	42	0	1.16 (0.78, 1.74)	14.84 (0.86, 257.19)
Avezzù *et al* (2008)	435	2	1	246	0	0	2.83 (0.14, 59.19)	1.70 (0.07, 41.84)
Cleary *et al* (2009) e	3697	63	11	2758	32	0	1.47 (0.96, 2.25)	17.16 (1.01, 291.31)
Lubbe *et al* (2009) f	9043	128	10	4962	75	0	0.94 (0.70, 1.25)	11.52 (0.68, 196.69)
*M–H pooled effect (fixed)*	25 231	350	35	18 285	236	0	1.07 (0.90, 1.26)	6.47 (2.33, 17.97)
*P*-value							0.44	<0.0005
*Heterogeneity*								
*P*-value							0.74	0.73
*I*^2^ (95% CI)							0 (0, 55)	0 (0, 68)

Abbreviations: CI=confidence interval; MM=mutated/mutated; NA=not available; OR=odds ratio; WM=wild type/mutant; WW=wild type/wild type.

a This table presents the data as they were published. Two unpublished studies included.

b Unpublished data obtained in 2006.

c Unpublished data obtained in 2007.

d Includes data from Farrington *et al* (2005), Tenesa *et al* (2006) and unpublished data from the SOCCS study obtained in 2008.

e Includes data from Croitoru *et al* (2004).

f Includes data from Webb *et al* (2006) and Fleischmann *et al* (2004).

**Table 5:** Meta-analysis of studies for the Y179C genotypes^a^

	**Y179C (cases)**			**Y179C (controls)**			**AG *vs* AA**	**GG *vs* AA**
**Study**	**AA**	**AG**	**GG**	**AA**	**AG**	**GG**	**OR (95% CI)**	**OR (95% CI)**
Enholm *et al* (2003)	994	1	0	424	0	0	1.28 (0.05, 31.50)	NA
Kambara *et al* (2004)	90	0	0	52	—	0	Not enough data available	NA
Wang *et al* (2004)	432	5	1	309	2	0	1.79 (0.35, 9.28)	2.15 (0.09, 52.87)
Zhou *et al* (2005)	432	3	0	466	2	0	1.62 (0.35, 9.28)	NA
Peterlongo *et al* (2005)	549	2	0	911	2	0	1.66 (0.23, 11.81)	NA
Tomlinson I (2006)^b^	662	6	0	197	0	0	3.88 (0.22, 69.10)	NA
Moreno *et al* (2006)	323	0	0	278	0	0	NA	NA
Colebatch *et al* (2006)	859	3	0	473	1	0	1.65 (0.17, 15.93)	NA
Koessler T (2007)^c^	2198	12	1	2204	11	0	1.09 (0.48, 2.48)	3.01 (0.12, 73.88)
Küry *et al* (2007)	999	3	0	1100	4	0	0.83 (0.18, 3.70)	NA
Balaguer *et al* (2007)	1089	4	2	912	1	0	3.35 (0.37, 30.02)	4.19 (0.20, 87.34)
SOCCS (2008)^d^	3429	21	3	2993	15	0	1.22 (0.63, 2.38)	6.11 (0.32, 118.34)
Avezzù *et al* (2008)	435	0	0	246	1	0	0.19 (0.01, 4.65)	NA
Cleary *et al* (2009) e	3697	15	5	2758	10	1	1.12 (0.50, 2.50)	3.73 (0.44, 31.95)
Lubbe *et al* (2009) f	9043	70	4	4962	26	1	1.48 (0.94, 2.32)	2.20 (0.25, 19.64)
*M-H pooled effect (fixed)*	25 231	145	16	18 285	75	2	1.34 (1.01, 1.77)	3.35 (1.14, 9.89)
*P*-value							0.04	0.03
								
*Heterogeneity*								
*P*-value							0.98	0.99
*I*^2^ (95% CI)							0 (0, 57)	0 (0, 75)

Abbreviations: CI=confidence interval; MM=mutated/mutated; NA=not available; OR=odds ratio; WM=wild type/mutant; WW=wild type/wild type.

a This table presents the data as they were published. Two unpublished studies included.

b Unpublished data obtained in 2006.

c Unpublished data obtained in 2007.

d Includes data from Farrington *et al* (2005), Tenesa *et al* (2006) and unpublished data from the SOCCS study obtained in 2008.

e Includes data from Croitoru *et al* (2004).

f Includes data from Webb *et al* (2006) and Fleischmann *et al* (2004).
