# Supplementary material for: A large-scale meta-analysis to refine colorectal cancer risk estimates associated with MUTYH variants
Source: Br J Cancer. 2010 Nov 9;103(12):1875–84. doi: 10.1038/sj.bjc.6605966 (PMC3008602; doi:10.1038/sj.bjc.6605966)
Supplement: Supplementary Material [file 6605966x1.doc]

Supplementary material

**Supplementary Box 1: Study Questionnaire**

**MYH Collaboration Questionnaire**

**A) Study details**

*Name of Study*

*Contact Investigator*

Name

Address

E-mail

Tel

Fax

*Contact for Data Extraction*

Name

E-mail

*Other investigators in this study participating in MYH Collaboration (max 2):*

Name 1

E-mail 1

Name 2

E-mail 2

*Key study reference considered relevant to this Collaboration (and to be quoted in subsequent publications*

…………………………………………………………………………………………………………

**B) Study data**

*Please name the MUTYH variant(s) that you have genotyped and tick the boxes of the data that you can provide:*

| **DATA** | **Variables** | **Description** | |
| --- | --- | --- | --- |
| **Genetic data** | *MUTYH* Variant(s)  Method of second allele analysis: | ………………………………  ……………………………… | |
|  |  |  | |
| **General data** | Age of diagnosis | |  |
|  | Gender | |  |
|  | Date of death | |  |
| **Clinical data** | How diagnosis verified | |  |
|  | Date of diagnosis | |  |
|  | Date of operation | |  |
|  | No of synchronous/ metachronous adenomas or adenocarcinomas | |  |
| **Demographic data** | No of first relatives to be affected | |  |
|  | No of second relatives to be affected | |  |
| **Controls** | Description of controls | |  |

**Supplementary Table 1: Data extraction table with 4** examples

| Subject Code | Case/ control status | MYH mut Y179C | MYH mut G396D | MYH mut other var | Genotype | | Sex | Ethnicity | Age at recruit | Date of Birth | Age of Diagn | Died | Date of death | Date of surgery | Histology | Grade | Location | Dukes stage | Sychron tumours/ polyps | Metachron tumours/ polyps | Histolog types | | No of polyps | | No of 1o relative affected | | No of 2o relative affected |
| --- | --- | --- | --- | --- | --- | --- | --- | --- | --- | --- | --- | --- | --- | --- | --- | --- | --- | --- | --- | --- | --- | --- | --- | --- | --- | --- | --- |
| 1452 | 1 | X | X |  | | MM | F | White | 72 | 12/06 /1930 | 71 | N |  | 25/05 /2001 | Adeno- carcinoma | II | Colon | A | 2 |  | | Tabular | | <3 | | 1 | 4 |
| 1234 | 1 |  | XX |  | | MM | M | Black | 59 | 14/02 /1948 | 58 | Y | 25/03 /2005 | 24/04 /2003 | Adeno- carcinoma | III | Rectum | B | 5 |  | | Tabular | | 3-100 | | 1 | 3 |
| 4567 | 0 |  |  |  | | WW | M | White | 50 | 25/10 /1945 |  | N |  |  |  |  |  |  |  |  | |  | |  | | 0 | 2 |
| 1657 | 0 | X |  |  | | WM | F | Black | 45 | 12/11 /1947 |  | N |  |  |  |  |  |  |  |  | |  | |  | | 0 | 1 |

Supplementary Table 2: Logistic regression analysis of the combined datasets; G396D analysis was conducted for individuals that were Y179C AA; Y179C analysis was conducted for individuals that were G396D GG; Combined genotype analysis was conducted for individuals with data for both Y179C and G396D.

|  |  |  | **Model II[[1]](#endnote-2)** | | | **Model III[[2]](#endnote-3)** | | |
| --- | --- | --- | --- | --- | --- | --- | --- | --- |
| **Gene** | **Cases** | **Controls** | **OR** | **95% CI** | **p-value** | **OR** | **95% CI** | **p-value** |
| **G396D**[[3]](#endnote-4) |  |  |  |  |  |  |  |  |
| *Whole sample* | | | | | | | | |
| GG | 19767 | 14723 | 1.00 |  |  | 1.00 |  |  |
| GA | 292 | 210 | 1.04 | 0.87, 1.25 | 0.67 | 1.04 | 0.87, 1.25 | 0.64 |
| AA | 31 | 0 | 23.68 | 3.23, 173.81 | 0.002 | 24.51 | 3.34, 180.08 | 0.002 |
| *≤55 years old* | | | | | | | | |
| GG | 6269 | 5270 | 1.00 |  |  | 1.00 |  |  |
| GA | 88 | 77 | 0.96 | 0.70, 1.31 | 0.80 | 0.96 | 0.70, 1.31 | 0.79 |
| AA | 13 | 0 | 11.41 | 1.49, 87.61 | 0.02 | 11.57 | 1.51, 88.95 | 0.02 |
| *>55 years old* | | | | | | | | |
| GG | 13498 | 9453 | 1.00 |  |  | 1.00 |  |  |
| GA | 204 | 133 | 1.08 | 0.86, 1.35 | 0.51 | 1.09 | 0.87, 1.36 | 0.44 |
| AA | 18 | 0 | 12.64 | 1.68, 94.99 | 0.01 | 13.02 | 1.73, 97.94 | 0.01 |
| *Males* | | | | | | | | |
| GG | 11229 | 6460 | 1.00 |  |  | 1.00 |  |  |
| GA | 161 | 95 | 0.99 | 0.76, 1.28 | 0.92 | 0.99 | 0.76, 1.28 | 0.93 |
| AA | 15 | 0 | 8.49 | 1.12, 64.27 | 0.04 | 9.17 | 1.20, 69.89 | 0.03 |
| *Females* | | | | | | | | |
| GG | 8528 | 8259 | 1.00 |  |  | 1.00 |  |  |
| GA | 131 | 115 | 1.09 | 0.85, 1.40 | 0.51 | 1.09 | 0.85, 1.40 | 0.51 |
| AA | 16 | 0 | 15.47 | 2.05, 116.70 | 0.008 | 15.45 | 2.05, 116.56 | 0.008 |

|  |  |  | **Model II[[4]](#endnote-5)** | | | **Model III[[5]](#endnote-6)** | | |
| --- | --- | --- | --- | --- | --- | --- | --- | --- |
| **Gene** | **Cases** | **Controls** | **OR** | **95% CI** | **p-value** | **OR** | **95% CI** | **p-value** |
| **Y179C[[6]](#endnote-7)** |  |  |  |  |  |  |  |  |
| *Whole sample* | | | | | | | | |
| AA | 19767 | 14723 | 1.00 |  |  | 1.00 |  |  |
| AG | 122 | 68 | 1.35 | 1.00, 1.82 | 0.05 | 1.33 | 0.99, 1.79 | 0.06 |
| GG | 11 | 2 | 3.97 | 0.88, 18.04 | 0.07 | 4.12 | 0.90, 18.81 | 0.07 |
| *≤55 years old* | | | | | | | | |
| AA | 6269 | 5270 | 1.00 |  |  | 1.00 |  |  |
| AG | 35 | 25 | 1.19 | 0.71, 2.00 | 0.52 | 1.18 | 0.70, 1.98 | 0.54 |
| GG | 9 | 0 | 6.44 | 0.81, 51.08 | 0.08 | 6.42 | 0.81, 51.07 | 0.08 |
| *>55 years old* | | | | | | | | |
| AA | 13498 | 9453 | 1.00 |  |  | 1.00 |  |  |
| AG | 87 | 43 | 1.46 | 1.01, 2.11 | 0.05 | 1.43 | 0.99, 2.07 | 0.06 |
| GG | 2 | 2 | 0.81 | 0.11, 5.79 | 0.83 | 0.84 | 0.11, 6.20 | 0.86 |
| *Males* | | | | | | | | |
| AA | 11229 | 6460 | 1.00 |  |  | 1.00 |  |  |
| AG | 68 | 23 | 1.68 | 1.04, 2.69 | 0.03 | 1.59 | 0.99, 2.57 | 0.06 |
| GG | 8 | 0 | 4.49 | 0.56, 35.92 | 0.16 | 4.17 | 0.52, 33.62 | 0.18 |
| *Females* |  |  |  |  |  |  |  |  |
| AA | 8528 | 8259 | 1.00 |  |  | 1.00 |  |  |
| AG | 54 | 45 | 1.15 | 0.77, 1.70 | 0.50 | 1.15 | 0.77, 1.70 | 0.50 |
| GG | 3 | 2 | 1.45 | 0.24, 8.70 | 0.68 | 1.45 | 0.24, 8.65 | 0.69 |

|  |  |  | **Model II[[7]](#endnote-8)** | | | **Model III[[8]](#endnote-9)** | | |
| --- | --- | --- | --- | --- | --- | --- | --- | --- |
| **Gene** | **Cases** | **Controls** | **OR** | **95% CI** | **p-value** | **OR** | **95% CI** | **p-value** |
| **Genotype[[9]](#endnote-10)** |  |  |  |  |  |  |  |  |
| *Whole sample* |  |  |  |  |  |  |  |  |
| WW | 19767 | 14723 | 1.00 |  |  | 1.00 |  |  |
| WM | 418 | 280 | 1.12 | 0.96, 1.31 | 0.16 | 1.12 | 0.96, 1.31 | 0.16 |
| MM | 76 | 2 | 29.77 | 7.30, 121.38 | 2.2x10^-6^ | 30.41 | 7.46, 124.02 | 1.92x10^-6^ |
| G396D AA | 31 | 0 | 23.68 | 3.23, 173.81 | 0.002 | 24.51 | 3.34, 180.08 | 0.002 |
| Y179C GG | 11 | 2 | 3.97 | 0.88, 18.04 | 0.07 | 4.12 | 0.90, 18.81 | 0.07 |
| Compound heterozygous[[10]](#endnote-11) | 29 | 0 | 23.02 | 3.13, 169.36 | 0.002 | 22.51 | 3.06, 165.62 | 0.002 |
|  |  |  |  |  |  |  |  |  |
| *≤55 years old* |  |  |  |  |  |  |  |  |
| WW | 6269 | 5270 | 1.00 |  |  | 1.00 |  |  |
| WM | 124 | 104 | 1.00 | 0.77, 1.31 | 0.98 | 1.00 | 0.77, 1.30 | 0.99 |
| MM | 43 | 0 | 36.08 | 4.96, 262.48 | 0.0004 | 36.12 | 4.96, 262.83 | 0.0004 |
| G396D AA | 13 | 0 | 11.41 | 1.49, 87.61 | 0.02 | 11.57 | 1.51, 88.95 | 0.02 |
| Y179C GG | 9 | 0 | 6.44 | 0.81, 51.08 | 0.08 | 6.42 | 0.81, 51.07 | 0.08 |
| Compound heterozygous | 17 | 0 | 13.99 | 1.85, 105.53 | 0.01 | 13.72 | 1.82, 103.55 | 0.01 |
|  |  |  |  |  |  |  |  |  |
| *>55 years old* |  |  |  |  |  |  |  |  |
| WW | 13498 | 9453 | 1.00 |  |  | 1.00 |  |  |
| WM | 294 | 176 | 1.18 | 0.98, 1.43 | 0.09 | 1.19 | 0.98, 1.44 | 0.08 |
| MM | 33 | 2 | 12.65 | 3.03, 52.86 | 0.001 | 13.09 | 3.13, 54.74 | 0.0004 |
| G396D AA | 18 | 0 | 12.64 | 1.68, 94.99 | 0.01 | 13.02 | 1.73, 97.94 | 0.01 |
| Y179C GG | 2 | 2 | 0.81 | 0.11, 5.79 | 0.83 | 0.84 | 0.11, 6.20 | 0.86 |
| Compound heterozygous | 12 | 0 | 9.60 | 1.24, 74.06 | 0.03 | 9.33 | 1.21, 72.09 | 0.03 |
|  |  |  |  |  |  |  |  |  |
| *Males* |  |  |  |  |  |  |  |  |
| WW | 11229 | 6460 | 1.00 |  |  | 1.00 |  |  |
| WM | 232 | 119 | 1.13 | 0.90, 1.41 | 0.29 | 1.12 | 0.89, 1.41 | 0.32 |
| MM | 36 | 0 | 20.28 | 2.78, 147.89 | 0.003 | 21.34 | 2.92, 156.05 | 0.003 |
| G396D AA | 15 | 0 | 8.49 | 1.12, 64.27 | 0.04 | 9.17 | 1.20, 69.89 | 0.03 |
| Y179C GG | 8 | 0 | 4.49 | 0.56, 35.92 | 0.16 | 4.17 | 0.52, 33.62 | 0.18 |
| Compound heterozygous | 12 | 0 | 6.76 | 0.88, 51.98 | 0.07 | 6.82 | 0.88, 52.83 | 0.07 |
|  |  |  |  |  |  |  |  |  |
| *Females* |  |  |  |  |  |  |  |  |
| WW | 8528 | 8259 | 1.00 |  |  | 1.00 |  |  |
| WM | 186 | 161 | 1.10 | 0.89, 1.37 | 0.36 | 1.10 | 0.89, 1.37 | 0.36 |
| MM | 40 | 2 | 19.48 | 4.71, 80.64 | 4.2x10^-5^ | 19.47 | 4.70, 80.58 | 4.2x10^-5^ |
| G396D AA | 16 | 0 | 15.47 | 2.05, 116.70 | 0.008 | 15.45 | 2.05, 116.56 | 0.008 |
| Y179C GG | 3 | 2 | 1.45 | 0.24, 8.70 | 0.68 | 1.45 | 0.24, 8.65 | 0.69 |
| Compound heterozygous | 17 | 0 | 16.50 | 2.19, 124.01 | 0.006 | 16.54 | 2.20, 124.35 | 0.006 |

**Supplementary Table 3: Mean age of onset analysis**

| ***Gene*** | ***Cases*** | | ***Controls*** | |  |
| --- | --- | --- | --- | --- | --- |
|  | **N** | **Age (SD)** | **N** | **Age (SD)** | **p-value^1^** |
| **MUTYH** |  |  |  |  |  |
| WW | 19774 | 59.6 (10.1) | 14495 | 59.1 (10.8) |  |
| WM | 416 | 59.9 (10.2) | 276 | 58.5 (10.8) |  |
| MM | 76 | 53.9 (8.3) | 2 | 60.5 (3.5) |  |
| *p-value^1^ for MUTYH* |  |  |  |  | <0.00005 |
| *p-value^1^ for status* |  |  |  |  | <0.00005 |
|  |  |  |  |  |  |
| **G396D** |  |  |  |  |  |
| GG | 19762 | 59.6 (10.1) | 14497 | 59.1 (10.8) |  |
| GA | 294 | 60.1 (10.6) | 206 | 58.9 (10.6) |  |
| AA | 31 | 56.7 (7.3) | 0 | n/a |  |
| *p-value^1^ for G396D* |  |  |  |  | 0.27 |
| *p-value^1^ for status* |  |  |  |  | <0.00005 |
|  |  |  |  |  |  |
| **Y179C** |  |  |  |  |  |
| AA | 19762 | 59.6 (10.1) | 14497 | 59.1 (10.8) |  |
| AG | 124 | 58.7 (9.3) | 68 | 57.4 (11.7) |  |
| GG | 11 | 48.9 (5.4) | 2 | 60.5 (3.5) |  |
| *p-value^1^ for Y179C* |  |  |  |  | 0.003 |
| *p-value^1^ for status* |  |  |  |  | <0.00005 |

^1^ Based on ANOVA

**Supplementary Table 4: Mean age of onset analysis**

|  | ***Cases*** | | | |  |
| --- | --- | --- | --- | --- | --- |
| **Gene** | **G396D** | | **Y179C** | |  |
|  | **N** | **Age (SD)** | **N** | **Age (SD)** | **p-value^1^** |
| **Genotype** |  |  |  |  |  |
| Wild type | 19762 | 59.6 (10.1) | 19762 | 59.6 (10.1) | n/a |
| Heterozygous | 294 | 60.1 (10.6) | 124 | 58.7 (9.3) | 0.19 |
| Variant | 31 | 56.7 (7.3) | 11 | 48.9 (5.4) | 0.003 |

^1^ Based on t-test

Supplementary Table 5: Interaction analysis between *MUTYH* variants and study population

| **Study** | **G396D** |  | **GG** |  | **GA** |  |
| --- | --- | --- | --- | --- | --- | --- |
|  | ***GG***  ***cases***  ***(controls)*** | ***GA***  ***cases***  ***(controls)*** | ***OR*** | ***95% CI*** | ***OR*** | ***95% CI*** |
| **Lubbe SJ** | 9043 (4962) | 128 (75) | 1.00 |  | 0.93 | 0.70, 1.24 |
| **Moreno V** | 336 (283) | 8 (7) | 0.60 | 0.51, 0.71 | 0.55 | 0.20, 1.54 |
| **Peterlongo P** | 552 (923) | 2 (5) | 0.37 | 0.33, 0.41 | 0.27 | 0.05, 1.40 |
| **Croitoru ME** | 1197 (1234) | 22 (17) | 0.52 | 0.48, 0.57 | 0.74 | 0.39, 1.40 |
| **Koessler T** | 2198 (2204) | 25 (31) | 0.61 | 0.57, 0.65 | 0.53 | 0.31, 0.92 |
| **Küry S** | 999 (1100) | 21 (16) | 0.46 | 0.42, 0.50 | 0.69 | 0.36, 1.33 |
| **SOCCS** | 3429 (2993) | 57 (42) | 0.60 | 0.57, 0.64 | 0.74 | 0.49, 1.11 |
| **Cleary SP** | 2013 (1024) | 32 (17) | 1.10 | 1.01, 1.20 | 0.96 | 0.53, 1.75 |
|  |  |  | p for interaction 0.75 | | | |
| **Study** | **Y179C** |  | **AA** |  | **AG** |  |
|  | ***AA***  ***cases***  ***(controls)*** | ***AG***  ***cases***  ***(controls)*** | ***OR*** | ***95% CI*** | ***OR*** | ***95% CI*** |
| **Lubbe SJ** | 9043 (4962) | 70 (26) | 1.00 |  | 1.53 | 0.97, 2.41 |
| **Moreno V** | 336 (283) | 0 (0) | 0.60 | 0.51, 0.71 | n/a |  |
| **Peterlongo P** | 552 (923) | 2 (2) | 0.37 | 0.33, 0.41 | 0.56 | 0.08, 4.03 |
| **Croitoru ME** | 1197 (1234) | 10 (4) | 0.52 | 0.48, 0.57 | 1.31 | 0.41, 4.23 |
| **Koessler T** | 2198 (2204) | 12 (11) | 0.61 | 0.57, 0.65 | 0.60 | 0.26, 1.36 |
| **Küry S** | 999 (1100) | 3 (4) | 0.46 | 0.42, 0.50 | 0.37 | 0.08, 1.66 |
| **SOCCS** | 3429 (2993) | 21 (15) | 0.60 | 0.57, 0.64 | 0.83 | 0.42, 1.61 |
| **Cleary SP** | 2013 (1024) | 6 (6) | 1.10 | 1.01, 1.20 | 0.56 | 0.18, 1.75 |
|  |  |  | p for interaction 0.52 | | | |
| **Study** | ***Genotype*** |  | **WW** |  | **WM** |  |
|  | ***WW***  ***cases***  ***(controls)*** | ***WM***  ***cases***  ***(controls)*** | ***OR*** | ***95% CI*** | ***OR*** | ***95% CI*** |
| **Lubbe SJ** | 9043 (4962) | 198 (101) | 1.00 |  | 1.08 | 0.85, 1.38 |
| **Moreno V** | 336 (283) | 8 (7) | 0.60 | 0.51, 0.71 | 0.55 | 0.20, 1.54 |
| **Peterlongo P** | 552 (923) | 4 (7) | 0.37 | 0.33, 0.41 | 0.36 | 0.10, 1.24 |
| **Croitoru ME** | 1197 (1234) | 29 (21) | 0.52 | 0.48, 0.57 | 0.75 | 0.43, 1.33 |
| **Koessler T** | 2198 (2204) | 37 (42) | 0.61 | 0.57, 0.65 | 0.55 | 0.35, 0.87 |
| **Küry S** | 999 (1100) | 25 (21) | 0.46 | 0.42, 0.50 | 0.61 | 0.34, 1.10 |
| **SOCCS** | 3429 (2993) | 77 (57) | 0.60 | 0.57, 0.64 | 0.75 | 0.53, 1.07 |
| **Cleary SP** | 2028 (1024) | 40 (24) | 1.11 | 1.02, 1.21 | 0.86 | 0.52, 1.44 |
|  |  |  | p for interaction 0.74 | | | |

Supplementary Table 6: Interaction analysis between *MUTYH* variants and HRT intake

| **HRT** | **G396D** |  | **GG** |  | **GA** |  | |
| --- | --- | --- | --- | --- | --- | --- | --- |
|  | ***GG***  ***cases***  ***(controls)*** | ***GA***  ***cases***  ***(controls)*** | ***OR*** | ***95% CI*** | ***OR*** | ***95% CI*** | |
| **yes** | 914 (958) | 13 (15) | 1.00 |  | 0.91 | 0.43, 1.92 | |
| **no** | 1492 (1282) | 29 (19) | 1.22 | 1.08, 1.37 | 1.60 | 0.89, 2.87 | |
|  |  |  | p for interaction 0.45 | | | | |
| **HRT** | **Y179C** |  | **AA** |  | **AG** |  | |
|  | ***AA***  ***cases***  ***(controls)*** | ***AG***  ***cases***  ***(controls)*** | ***OR*** | ***95% CI*** | ***OR*** | ***95% CI*** | |
| **yes** | 914 (958) | 6 (2) | 1.00 |  | 3.14 | 0.63, 15.62 | |
| **no** | 1492 (1282) | 9 (10) | 1.22 | 1.08, 1.37 | 0.94 | 0.38, 2.33 | |
|  |  |  | p for interaction 0.12 | | | | |
| **HRT** | ***Genotype*** |  | **WW** |  | **WM** |  | |
|  | ***WW***  ***cases***  ***(controls)*** | ***WM***  ***cases***  ***(controls)*** | ***OR*** | ***95% CI*** | ***OR*** | ***95% CI*** | |
| **yes** | 918 (957) | 17 (18) | 1.00 |  | 0.98 | 0.50, 1.92 | |
| **no** | 1492 (1282) | 37 (29) | 1.21 | 1.08, 1.37 | 1.33 | 0.81, 2.18 | |
|  |  |  | p for interaction 0.80 | | | |  |

Supplementary Figure 1: Meta-analysis of studies comparing *MUTYH* G396D AA vs. GG.

(SOCCS data include: Farrington SM (published in 2005), Tenesa A (published in 2006) and unpublished data from the SOCCS study obtained in 2008; Cleary SP data include Croitoru ME (published in 2004); Lubbe SJ data include Webb EL (published in 2006) and Fleischmann C (published in 2005)).

Supplementary Figure 2: Meta-analysis of studies comparing *MUTYH* G396D GA vs. GG.

(SOCCS data include: Farrington SM (published in 2005), Tenesa A (published in 2006) and unpublished data from the SOCCS study obtained in 2008; Cleary SP data include Croitoru ME (published in 2004); Lubbe SJ data include Webb EL (published in 2006) and Fleischmann C (published in 2005)).

Supplementary Figure 3: Meta-analysis of studies comparing *MUTYH* Y179C GG vs. AA.

(SOCCS data include: Farrington SM (published in 2005), Tenesa A (published in 2006) and unpublished data from the SOCCS study obtained in 2008; Cleary SP data include Croitoru ME (published in 2004); Lubbe SJ data include Webb EL (published in 2006) and Fleischmann C (published in 2005)).

Supplementary Figure 4: Meta-analysis of studies comparing *MUTYH* Y179C AG vs. AA.

(SOCCS data include: Farrington SM (published in 2005), Tenesa A (published in 2006) and unpublished data from the SOCCS study obtained in 2008; Cleary SP data include Croitoru ME (published in 2004); Lubbe SJ data include Webb EL (published in 2006) and Fleischmann C (published in 2005)).

**Supplementary Figure 5:** Funnel Plot for Homozygous (MM vs. WW)

The funnel plot appears asymmetric, with large studies showing no effect missing (middle left part of graph). The reasons for this asymmetry might be due to (Egger et al 1997):

(i) Selection bias (publication bias, location bias, language bias, citation bias, multiple publications bias)

(ii) True heterogeneity (size of effect differs according to study size)

(iii) Data irregularities (poor methodological design of small studies, inadequate analysis, fraud)

(iv) Artifact (heterogeneity due to poor choice of effect measure)

(v) Chance

To test for the significance of the funnel plot asymmetry, the Harbord test was used as recommended in the Cochrane Handbook for systematic Reviews of Interventions.

P for small study effect = 0.31

**Supplementary Figure 6:** Funnel Plot for Heterozygous (WM vs. WW)

The funnel plot appears asymmetric, with small studies showing an inverse association missing (middle and bottom left part of graph).

Harbord test for small study effect: p = 0.26

1. Analysis adjusted for age and sex [↑](#endnote-ref-2)
2. Analysis adjusted for age, sex and study [↑](#endnote-ref-3)
3. Analysis conducted only for the AA Y179C ie. WW [↑](#endnote-ref-4)
4. Analysis adjusted for age and sex [↑](#endnote-ref-5)
5. Analysis adjusted for age, sex and study [↑](#endnote-ref-6)
6. Analysis conducted only for the GG G396D ie. WW [↑](#endnote-ref-7)
7. Analysis adjusted for age and sex [↑](#endnote-ref-8)
8. Analysis adjusted for age, sex and study [↑](#endnote-ref-9)
9. Including subjects with data for both Y179C and G396D [↑](#endnote-ref-10)
10. This category includes 29 G396D GA and Y179C AG cases; 5 cases with either G396D GA or Y179C AG and an other pathogenic *MUTYH* mutation were excluded [↑](#endnote-ref-11)
